# Calcium titanate nanoparticles-induced cytotoxicity, genotoxicity and oxidative stress in human non-small lung cancer cells

**DOI:** 10.1038/s41598-025-89035-8

**Published:** 2025-02-21

**Authors:** Hanan R. H. Mohamed, Shahd E. E. Shaheen, Esraa H. Ibrahim, Nesma O. E. Hussein, Gehan Safwat

**Affiliations:** 1 Zoology Department Faculty of Science, Cairo University, Giza, Egypt; 2 Faculty of Biotechnology, October University for Modern Sciences and Arts, Giza, Egypt

**Keywords:** Calcium titanate nanoparticles, Genotoxicity, Cytotoxicity, Oxidative stress, Apoptosis, Lung cancer, Molecular biology, Biomarkers, Risk factors

## Abstract

Calcium titanate nanoparticles (CaTiO_3_NPs) have garnered significant attention due to their unique properties and excellent biocompatibility, which have led to their increased use in various fields and consumer products. This rise in application necessitates a better understanding of their biological and toxicological effects. However, there is limited data on the cytotoxicity and genotoxicity of CaTiO_3_NPs in human normal skin fibroblasts (HSF) and non-small lung cancer (A-549) cells. Consequently, this study aimed to explore the effect of 48-hour exposure to CaTiO_3_NPs on cell viability, genomic DNA integrity, and oxidative stress induction in human cancer A-549 cells, compared to normal HSF cells. The cytotoxicity and genotoxicity of CaTiO_3_NPs were assessed using the Sulforhodamine B (SRB) cytotoxicity and Alkaline Comet assays, respectively. To estimate possible oxidative stress induction and variation in apoptotic gene expression, reactive oxygen species (ROS) analysis and quantitative real-time polymerase chain reaction (qRT-PCR) were also performed. Our findings demonstrated that exposure to CaTiO_3_NPs for 48 h resulted in low toxicity toward both normal HSF and cancer A-549 cells, with cell death observed only at high concentrations (100 and 1000 µg/ml). The IC50 value of CaTiO_3_NPs in both HSF and A-549 cells was greater than 1000 µg/ml; specifically, the IC50 value in A-549 cells at 48 h was 1670.65 µg /ml. However, treatment with CaTiO_3_NPs for 48 h at the IC50 concentration of 1670.65 µg /ml resulted in significant genomic DNA damage and excessive ROS generation, along with a notable disturbance in the expression level of apoptotic (p53 and Bax) and anti-apoptotic Bcl2 genes in A-549 cells. In contrast, no significant changes were observed in HSF cells treated for 48 h with the same concentration (1670.65 µg /ml) of CaTiO_3_NPs. Collectively, these findings indicated that despite short-term exposure to CaTiO_3_NPs causing low cytotoxicity in both normal HSF and A-549 cells. CaTiO_3_NPs were selectively genotoxic toward A-549 cells. This genotoxicity was mediated through excessive ROS generation, which disrupted genomic DNA integrity and altered the expression of apoptotic genes, triggering apoptosis in A-549 cells. Further *in vitro and in vivo* studies are needed to fully understand the toxicological and biological properties of CaTiO_3_NPs.

## Introduction

In today’s world, the prevalence of nanoparticles in various industries is increasing due to their unique properties and applications^1,2^. One type of nanoparticle that has gained significant attention is calcium titanate nanoparticles (CaTiO_3_NPs), a class of ceramic nanoparticles. These nanoparticles are tiny particles composed of calcium and titanium oxides, and are synthesized through various methods, including sol-gel, hydrothermal, and co-precipitation techniques, each offering distinct advantages depending on the desired application^1,3–5^.

CaTiO_3_NPs have garnered interest in the scientific community due to their exceptional electrical, optical, and catalytic properties^3–5^. These properties make CaTiO₃NPs highly versatile and applicable across a wide range of industries, including materials science, electronics energy storage, catalysis, and even medical imaging, pharmaceutics, paints and water treatment. Their unique properties, such as biocompatibility, high surface area, piezoelectricity, and photocatalytic activity, make them valuable tools in advancing technologies for drug delivery, cancer therapy, environmental cleanup, and energy storage. With continued research into their potential, CaTiO_3_NPs are expected to play a significant role in both industrial and healthcare applications, offering innovative solutions to current challenges^3–7^.

Recently CaTiO_3_NPs exhibited a promising potent cytotoxic and selective genotoxic effects against human breast cancer MCF-7 cells through inhibition of cancer cell proliferation and induction of excessive reactive oxygen species (ROS) generation that disrupts the genomic DNA stability triggering apoptosis of human breast cancer MCF-7 cells^8^. However, no research has yet explored the effects of CaTiO_3_NPs on cancer cell types other than breast cancer MCF-7 cells, particularly in lung cancer A-549 cells.

Investigating the cytotoxicity and genotoxicity of CaTiO_3_NPs on normal human cells is crucial for understanding their potential impact on human health, especially given their applications in various fields. The study by Mohamed et al.^9^. found that while exposure to CaTiO_3_NPs for 72 h resulted in the death of human HSF cells, the integrity of genomic DNA, levels of ROS generation, and expression of apoptotic genes remained normal.

The lack of adequate data on the cytotoxicity and genotoxicity of CaTiO_3_NPs across different human cell lines and time points highlights the need for further research. This is essential to thoroughly evaluate the effects of these nanoparticles on both normal and cancer cells before their widespread use, ensuring safety and minimizing unintended consequences. Consequently, the current study was undertaken to estimate the effect of CaTiO_3_NPs on cell proliferation, genomic DNA integrity and oxidative stress induction in human normal skin fibroblasts (HSF) and non-small cell lung cancer (A549) cell lines.

The innovations of this study lies in exploring the selective genotoxicity of CaTiO_3_NPs in cancerous A-549 lung cells, particularly in relation to oxidative stress and DNA damage. While previous studies have examined the general toxicity of nanoparticles, there is limited understanding of how CaTiO_3_NPs specifically induce oxidative stress and DNA damage in cancer cells, while potentially sparing normal cells. In addition, this study aims to provide valuable information on the mechanisms of CaTiO_3_NPs-induced genotoxicity.

The effect of CaTiO_3_NPs on viability of HSF and A-549 cells was studied using Sulforhodamine B (SRB) cytotoxicity assay, while, Alkaline Comet assays were performed to measure the level of induced genomic DNA damage in both HSF and A-549 cells. The level of ROS generation within HSF and A-549 cells was analyzed using 2,7-dichlorofluorescein diacetate dye, and also the expression level of apoptosis related genes was measured in untreated and treated HSF and A-549 cells using quantitative real time polymerase chain reaction (qRT-PCR).

## Materials and methods

### Chemicals

CaTiO_3_NPs were obtained from Sigma-Aldrich Chemical Company (Saint Louis, USA) in the form of white powders and product number 633,801 and purity 99% trace metals basis. Prior use powders of CaTiO_3_NPs were ultra-sonicated and suspended in Dimethyl Sulfoxide (DMSO) with final concentration ≤ 0.1% to prepare the tested concentrations. Bovine Serum Albumin (BSA) with Fraction V ≥ 99%, DMSO (CAS No. 67-68-5) and Sulforhodamine B (SRB) ≥ 85% was purchased from Sigma-Aldrich (St. Louis, MO, USA) were sourced from Sigma-Aldrich (St. Louis, MO, USA), while Dulbecco’s Modified Eagle Medium (DMEM) with glucose and without phenol red was obtained from Gibco (Thermo Fisher Scientific, Waltham, MA, USA).

### Characterization of CaTiO3NPs

Characterization of CaTiO_3_NPs using X-ray diffraction (XRD) analysis, transmission electron microscope (TEM) and Malvern Instrument Zeta sizer Nano Series (Malvern Instruments, Westborough, MA) has been previously conducted^8,9^ to ensure the purity, nano-size and well distribution of these nanoparticles in aqueous media. For the characterization of CaTiO_3_NPs using the Malvern Zetasizer Nano Series and TEM, a suspension was prepared by dispersing the CaTiO_3_NPs powder in deionized distilled water. The dispersion was achieved using an ultrasonic bath to ensure uniform distribution of the nanoparticles.

### Human cell lines

Normal Human Skin Fibroblast (HSF) and Non-Small Cell Lung Cancer (A-549) cells were obtained from Nawah Scientific Company (Mokatam Cairo Egypt) and were maintained in Dulbecco’s Modified Eagle Medium (DMEM) media supplemented with 100 mg/ml of streptomycin, 100 units/mL of penicillin and 10% of heat-inactivated fetal bovine serum in humidified, 5% (v/v) CO_2_ atmosphere at 37 °C.

### Cytotoxicity sulforhodamine B (SRB) assay

Impact of CaTiO_3_NPs on to viability of normal HSF and cancer A-549 cells was assessed using SRB cytotoxicity assay^10^. Aliquots of 100 µl cell suspension (5 × 10^^3^ cells) were cultured in 96-well plates and incubated in complete media for 24 h. Cells were then treated with another aliquot of 100 µl media containing CaTiO_3_NPs at various concentrations (0.1, 1, 10, 100 and 1000 µg/ml for both HSF and A-549 cells) or (1, 10, 100, 1000 and 10000 µg/ml for only A-549 cells). To mitigate nanoparticle aggregation and settling over time, we made sure to periodically agitate the suspension during the cell treatment process to maintain uniform dispersion in the well plates. After 48 h of nanoparticles exposure, HSF and A-549 cells were fixed by replacing media with 150 µl of 10% Trichloroacetic acid (TCA) (Sigma-Aldrich St. Louis, MO, USA) and incubated at 4 °C for 1 h. The TCA solution was removed, and the cells were washed 5 times with distilled water. Aliquots of 70 µl SRB solution (0.4% w/v) were added and incubated in a dark place at room temperature for 10 min. Plates were washed 3 times with 1% acetic acid and allowed to air-dry overnight. Then, 150 µl of TRIS (10 mM) was added to dissolve protein bound SRB stain; the absorbance was measured at 540 nm using an Infinite F50 microplate reader (TECAN, Switzerland).

### Treatment design

Normal HSF and lung cancer A-549 cells were cultured under the proper conditions and divided into control and treated cells. Control cells were treated with an equal volume of the vehicle (DMSO; final concentration, ≤ 0.1%), while treated cells were treated with the determined IC50 of CaTiO_3_NPs. After 48 h of nanoparticles treatment, untreated and treated cells were harvested by trypsinization and centrifugation. Each treatment was done in triplicate, and cells were washed twice with ice-cold PBS and stored at -80 C° for different molecular studies.

### Alkaline Comet assay

The impact of CaTiO_3_NPs on genomic DNA integrity was assessed in both treated and untreated HSF and A-549 cells using the Alkaline Comet assay^11,12^. A 15 µl aliquot of cell suspension was mixed with 60 µl of low melting agarose, thoroughly mixed well and then spread on slides pre-coated with normal melting agarose. After the gel had hardened, the slides were immersed in a lysis buffer with a pH of 10, containing freshly added Triton X-100 and Dimethyl sulfoxide (DMSO), and incubated for 24 h in the dark. Following lysis, the slides were washed with distilled water and incubated in an alkaline electrophoresis buffer with a pH greater than 12 to unwind the double-stranded DNA. Electrophoresis was then conducted for 30 min at a current of 300 mA and 35 V. After electrophoresis, pH neutralization was performed using Tris buffer to reanneal the single-stranded DNA. The DNA was then fixed with absolute ethanol for permanent preservation. Finally, the slides were stained with ethidium bromide (Sigma-Aldrich, St. Louis, MO, USA), and images were captured using an epi-fluorescent microscope (Leica Microsystems, Wetzlar, Germany) at a magnification of 200×. Fifty comet nuclei from each sample were analyzed using Comet Score™ software.

### Production of intracellular ROS

The effect of CaTiO_3_NPs on the generation of reactive oxygen species (ROS) within HSF and A-549 cells was also screened using 2,7 dichlorofluorescein diacetate dye that enters cells passively and reacts with ROS forming the highly fluorescent dichlorofluorescein product^13^. A cell suspension was mixed with 2,7-dichlorofluorescein diacetate dye (Sigma-Aldrich St. Louis, MO, USA) and incubated in dark for 30 min. After incubation this mixture was spread on a clean slide and the emitted fluorescent light was validated using epi-fluorescent (Leica Microsystems, Wetzlar, Germany) at 200 × magnification. The epi-fluorescent microscope is used to detect fluorescence from 2,7-dichlorofluorescein diacetate dye, with an excitation wavelength of 485 nm and an emission wavelength of 530 nm. This allows the detection of the green fluorescence emitted by 2,7-dichlorofluorescein diacetate upon oxidation by ROS.

### Quantitative real time PCR

Influence of CaTiO_3_NPs on the mRNA expression level of p53, Bax and Bcl2 genes in the untreated and treated HSF and A549 cells was studied using Quantitative Real Time Polymerase Chain Reaction (qRTPCR). The GeneJET RNA Purification Kit (Thermo scientific, USA) was first used to extract the total cellular RNA that was converted subsequently into complementary DNA (cDNA) using the Revert Aid First Strand cDNA Synthesis Kit (Thermo scientific, USA). The obtained cDNA of p53, Bax and Bcl2 genes were finally amplified using SYBR Green master mix and the primers sequence shown in Table 1^14,15^ by the 7500 Fast system (Applied Biosystem 7500, Clinilab, Egypt). The mRNA expression level of the studied apoptotic and anti-apoptotic genes was then determined using the comparative Ct (DDCt) method after standardization using the housekeeping GAPDH gene expression. The final results were expressed as mean ± S.D.

**Table 1:** Sequences of the used primers in qRT-PCR.

Gene	Strand	Primer’s sequences
GAPDH	Forward	5ʹ-GAAGGTGAAGGTCGGAGTCA-3ʹ
	Reverse	5ʹ-GAAGATGGTGATGGGATTTC-3ʹ
BAX	Forward	5ʹ-CCGCCGTGGACACAGAC-3ʹ
	Reverse	5ʹ-CAGAAAACATGTCAGCTGCCA-3ʹ
BCL2	Forward	5ʹ-TCCGATCAGGAAGGCTAGAGT-3ʹ
	Reverse	5ʹ-TCGGTCTCCTAAAAGCAGGC-3ʹ
P53	Forward	5ʹ-CAGCCAAGTCTGTGACTTGCACGTAC-3ʹ
	Reverse	5ʹ-CTATGTCGAAAAGTGTTTCTGTCATC-3ʹ

### Statistical analysis

The obtained results were displayed as mean ± Standard Deviation (S.D) and were analyzed using the Statistical Package for the Social Sciences (SPSS) (version 20) at the significance level *p* > 0.05. One Way Analysis of Variance (ANOVA) followed by Duncan’s test was done to compare among the untreated and treated normal HSF and lung cancer A-549 cells.

## Results

### Characterization of CaTiO3NPs

In a study conducted by Mohamed and her colleagues^8,9^, the purity of CaTiO_3_NPs was confirmed through XRD analysis, which revealed distinctive peaks at theta angles of 32.7º, 47.1°, 58.9°, and 69°. Further analysis using a Zeta sizer analyzer and transmission electron microscope showed that CaTiO_3_NPs were well distributed and exhibited a spherical shape in aqueous media. The mean Zeta potential value was determined to be -3.38 mv, with an average particle size of 88.79 ± 22.32 nm.

### Effect of CaTiO3NPs on cell viability

**Fig. 1:**
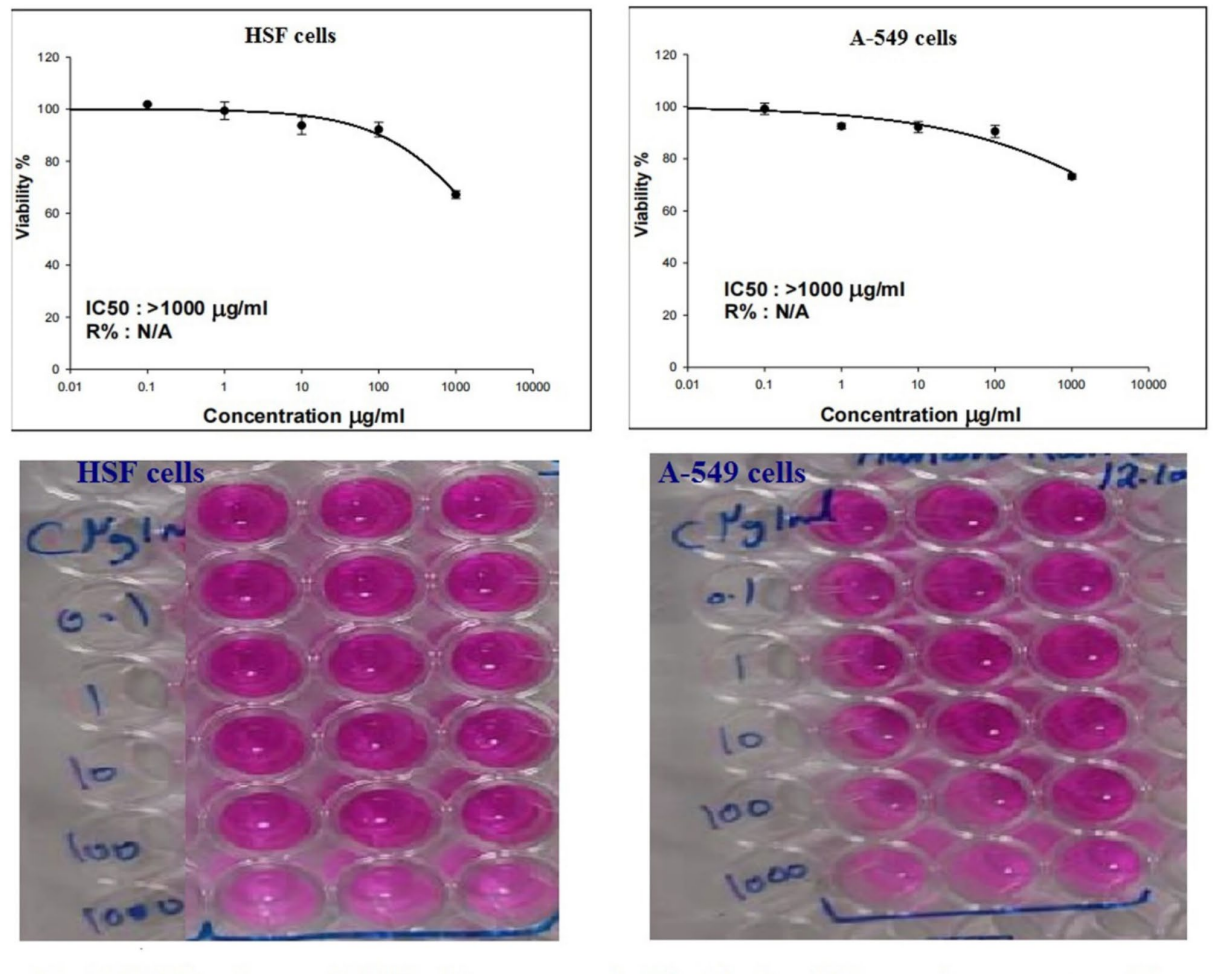
Viability of normal HSF and lung cancer A-549 cells after 48 h of exposure to CaTiO_3_NPs. Results are expressed as mean. Triplicates were done for each concentration.

Interpretation of cytotoxicity SRB assay data mirrored the low toxicity of CaTiO_3_NPs on both normal HSF and lung cancer A-549 cells. As depicted in Fig. 1 treatment of normal HSF and lung cancer A-549 cells with CaTiO_3_NPs five concentrations: 0.1, 1, 10, 100 and 1000 µg/ml for 48 h did not affect cell viability and high cell death was observed in HSF and A-549 cells treated with 1000 µg/ml of CaTiO_3_NPs, and so the half maximal inhibitory concentration (IC50) of CaTiO_3_NPs was greater than 1000 µg/ml in both normal HSF and lung cancer A-549 cells (Fig. 1).

**Fig. 2:**
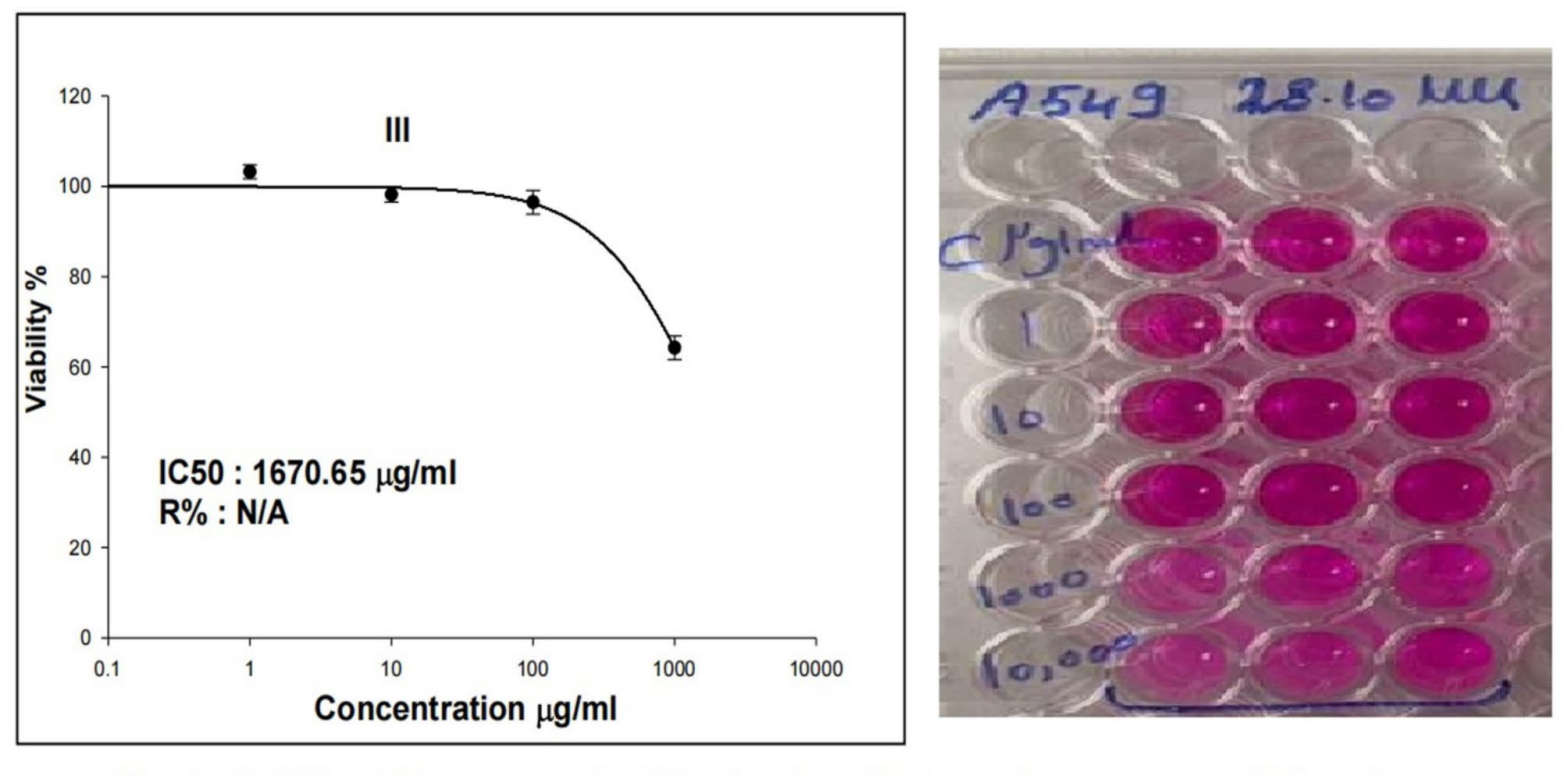
Viability of lung cancer A-549 cells after 48 h of exposure to CaTiO_3_NPs. Results are expressed as mean. Triplicates were done for each concentration.

Similarly, treatment of lung cancer A-549 cells with CaTiO_3_NPs at five concentrations 1, 10, 100, 1000 and 10,000 µg/ml for 48 h markedly did not affect cell viability at low concentrations (1, 10 and 100 µg/ml) and highly decreased A-549 cells viability at high concentrations (1000 and 10000 µg/ml) in a concentration dependent manner, thus the IC50 value for CaTiO_3_NPs was 1670.65 µg/ml in lung cancer A-549 cells (Fig. 2). Consequently, both of normal HSF and lung cancer A-549 cells were treated with the same IC50 value of CaTiO_3_NPs that is 1670.65 µg /ml for subsequent studies.

### Effect of CaTiO3NPs on genomic DNA integrity

**Fig. 3:**
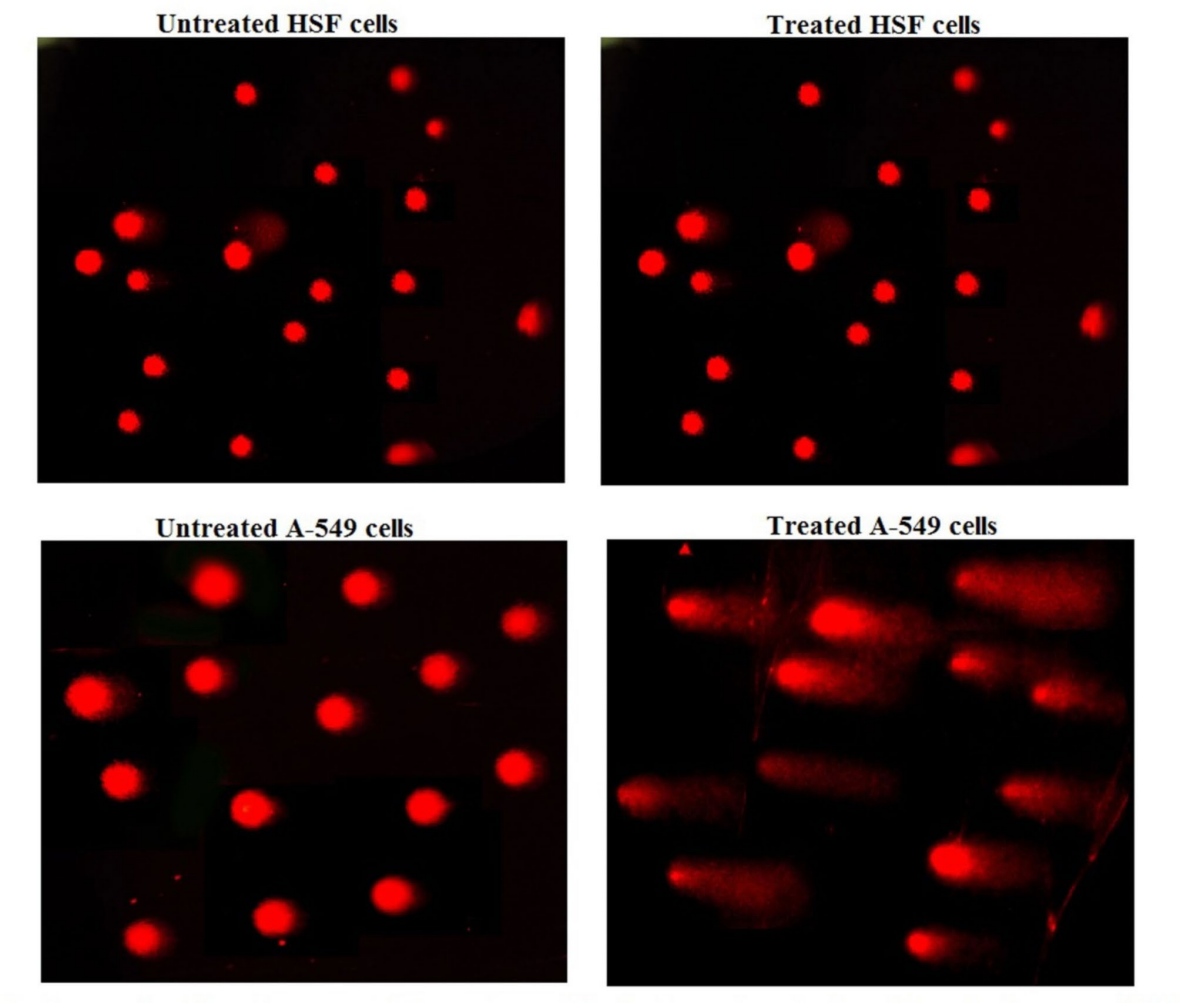
Examples for the scored Comet nuclei of the untreated and treated normal HSF and lung cancer A-549 cells with IC50 of CaTiO_3_NPs for 48 h.

Results of alkaline Comet assay demonstrated the selective genotoxicity of CaTiO_3_NPs towards cancerous genomic DNA. This was manifested from the non-significant variations in the comet parameters: tail length, %DNA in tail and tail moment noticed after 48 h of normal HSF cells treatment with CaTiO_3_NPs (1670.65 µg/ml) compared to their values in the untreated HSF cells (Table 2; Fig. 3). On contrary, remarkable increases in tail length, %DNA in tail and tail moment were seen after 48 h of lung cancer A-549 treatment with the same concentration of CaTiO_3_NPs (1670.65 µg/ml) compared to the untreated HSF and A-549 cell values (Table 2; Fig. 3).

**Table 2:** Level of DNA damage induction in normal HSF and cancerous lung A-549 cells treated with IC50 of CaTiO_3_NPs for 48 h.

Cells	CaTiO_3_NPs concentration	Tail length (px)	%DNA in tail	Tail moment
Control HSF cells	0.00	3.45 ± 0.25^a^	15.57 ± 1.88^a^	0.67 ± 0.09^a^
Treated HSF cells	1670.65 µg/ml	3.05 ± 0.31^a^	16.56 ± 2.32^a^	0.53 ± 0.04^a^
Control lung A-549 cells	0.00	4.01 ± 0.75^a^	17.03 ± 3.00^b^	0.76 ± 0.31^a^
Treated lung A-549 cells	1670.65 µg/ml	11.23 ± 1.75^b^	29.15 ± 2.71^c^	3.17 ± 0.46^b^

Results are expressed as mean ± SD.

Results were analyzed using one-way analysis of variance followed by Duncan’s test to test the similarity between the control and treated HSF and lung cancer A-549 cells.

Means with different letters indicates statistical significant difference at *p* < 0.05 between the compared cells in the same column.

### Effect of CaTiO3NPson ROS generation

**Fig. 4:**
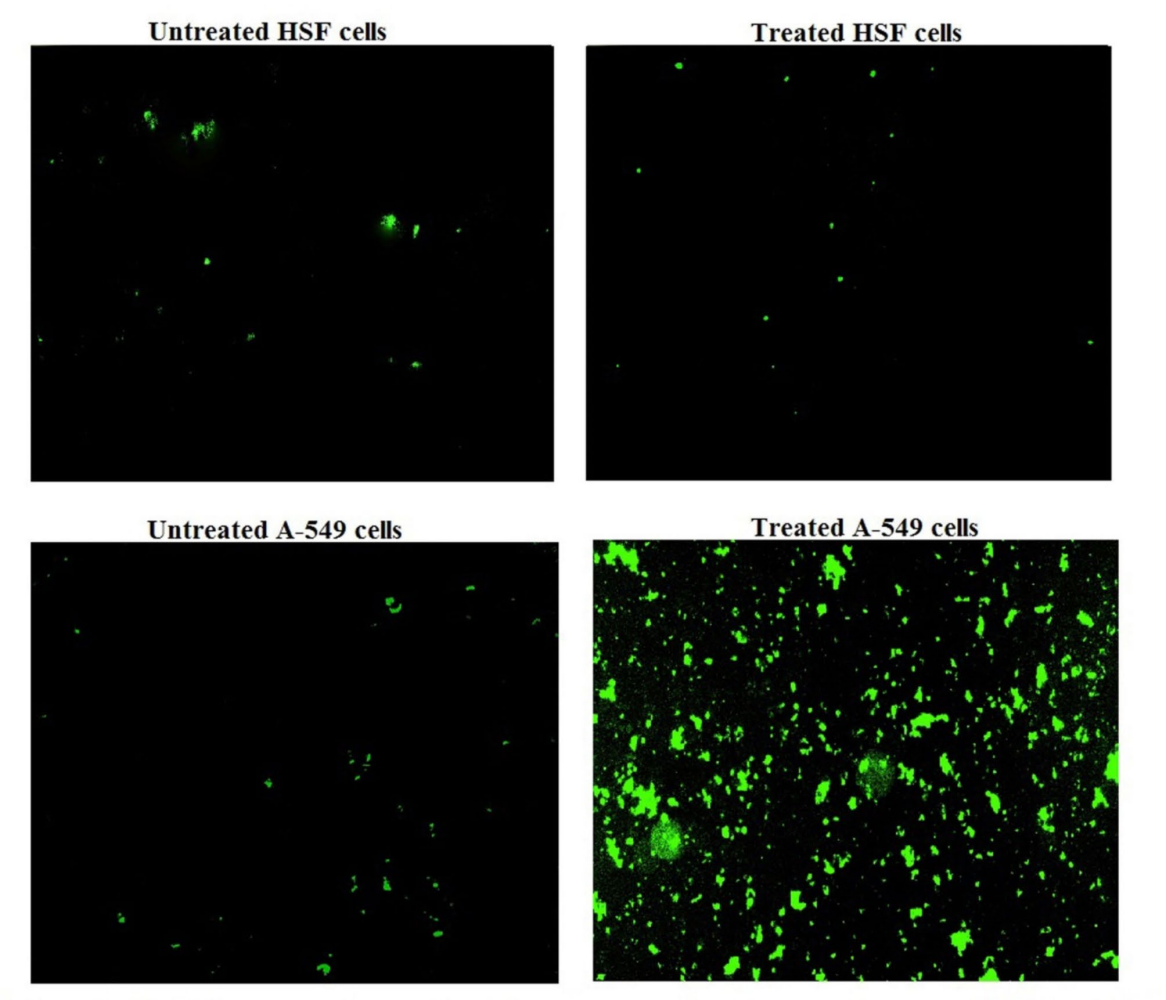
Level of ROS generation within the untreated and treated normal HSF and lung cancer A-549 cells with IC50 of CaTiO_3_NPsfor 48 h.

Using 2,7-dichlorofluorescein diacetate dye it was found that exposure of normal HSF to a 1670.65 µg/ml of CaTiO_3_NPs for 48 did not cause any remarkable change in the generation level of intracellular ROS compared to the untreated HSF generation level (Fig. 4). However, treatment of cancerous lung A-549 cells with the same concentration of CaTiO_3_NPs (1670.65 µg/ml) caused significant elevations in the ROS generation as depicted from remarkable increases in the emitted fluorescent light intensity compared to that emitted from the untreated A-549 cells (Fig. 4).

### Effect of CaTiO3NPs on apoptotic and anti-apoptotic gene expression

As shown in Table 3 treatment of normal HSF cells with CaTiO_3_NPs at a concentration of 1670.65 µg/ml caused a remarkable elevation only in the expression level of anti-apoptotic Bcl2 gene but no significant variations were noticed in the expression level of apoptotic p53 and Bax genes compared to their expression level in the untreated normal HSF cells. Meanwhile, the expression level of apoptotic p53 and Bax genes was significantly down regulated and the expression level of anti-apoptotic Bcl2 gene was highly upregulated in the untreated lung cancer cells compared to their expression level in the untreated HSF cells (Table 3).

**Table 3:** Fold change in the expression level of p53, BAX and BCL2 genes in normal HSF and cancerous lung A-549 cells treated with IC50 of CaTiO_3_NPs for 48 h.

Cells	CaTiO_3_NPs concentration	p53 gene	BAX gene	BCL2 gene
Control HSF cells	0.00	1.00 ± 0.00^a^	1.00 ± 0.00^a^	1.00 ± 0.00^a^
Treated HSF cells	1670.65 µg/ml	0.95 ± 0.03^a^	1.09 ± 0.08^a^	1.77 ± 0.09^b^
Control lung A-549 cells	0.00	0.28 ± 0.05^b^	0.17 ± 0.05^b^	3.11 ± 0.12^c^
Treated lung A-549 cells	1670.65 µg/ml	4.47 ± 0.74^c^	8.60 ± 1.15^c^	0.44 ± 0.06^d^

Results are expressed as mean ± SD.

Results were analyzed using one-way analysis of variance followed by Duncan’s test to test the similarity between the control and treated HSF and lung cancer A-549 cells.

Means with different letters indicates statistical significant difference at *p* < 0.05 between the compared cells in the same column.

On the other hand, remarkable elevations in the expression level of apoptotic p53 and Bax genes along with significant decreases in the expression level of anti-apoptotic Bcl2 gene were seen after 48 h of lung cancer A-549 cells treatment with CaTiO_3_NPs (1670.65 µg/ml) compared to their expression level in the untreated A-549 cells (Table 3).

## Discussion

CaTiO₃NPs) are currently gaining considerable attention for their unique properties and potential applications across various fields, alongside recent findings of their strong cytotoxic and genotoxic effects against human breast cancer MCF-7 cells^8,9^. However, their cytotoxicity and genotoxicity in human normal and lung cancer cells over a 48-hour exposure period have not been thoroughly investigated. Therefore, this study was conducted to assess the cytotoxicity, genomic DNA damage, and oxidative stress induced by CaTiO₃NPs in human normal HSF and lung cancer A-549 cells at 48 h.

To understand the cytotoxic risk associated with CaTiO_3_NPs, cytotoxicity SRB assay was conducted. Results of cytotoxicity assay has shown that exposure to CaTiO_3_NPs for 48 h was low toxic to normal HSF and lung cancer A-549 cells and caused cell death only at high concentrations (100 and 1000 µg/ml) and thus IC50 of CaTiO_3_NPs was found greater than 1000 µg/ml in HSF and A-549 cells. Consistency with our results no remarkable changes were observed in viability and proliferation of normal HSF and breast cancer MCF-7 cells after 24 h of cells treatment with CaTiO_3_NPs^8,9^.

In addition to cytotoxicity, another major area of concern is the genotoxicity of CaTiO_3_NPs. Genotoxicity refers to the ability of a substance to cause damage to the genetic material within cells, potentially leading to mutations and other harmful effects^16,17^. The results of the alkaline Comet assay demonstrated the selective genotoxicity of CaTiO_3_NPs towards cancer A-549 lung cells. Exposure to these nanoparticles caused significant disruption of genomic stability, leading to marked increases in DNA damage parameters, including tail length, %DNA in tail and tail moment only in the treated cancer A-549 lung cells. In contrast, no significant changes in these parameters were observed in the treated HSF cells compared to the untreated cells. These findings are consistent with recent studies that reported selective DNA damage induction in CaTiO_3_NPs-treated MCF-7 breast cancer cells, while showing no significant alteration in the genomic integrity of CaTiO_3_NPs treated HSF cells^6,7^.

One of the primary mechanisms through which nanoparticles induce genotoxicity is the induction of oxidative stress. Oxidative stress occurs when the production of reactive cellular ROS exceeds the capacity of the cell’s antioxidant defense systems, leading to direct DNA damage and genomic instability. This oxidative damage offers a therapeutic opportunity for selectively inducing DNA damage in cancer cells^18,19^. Selective oxidative stress induction by CaTiO_3_NPs in cancerous A-549 lung cells was manifested by the remarkable elevations noticed in the ROS generation level within CaTiO_3_NPs treated A-549 lung cells conversely to the non-observable variations in ROS generation level noticed in CaTiO_3_NPs treated HSF cells compared to the untreated cells.

The observed non-significant ROS generation between untreated and treated HSF cells, alongside significant ROS generation in cancer A-549 lung cells shown in Fig. 4, could be explained by the inherent differences in oxidative stress responses between normal and cancerous cells. Cancer cells often have altered metabolic pathways and upregulated antioxidant defenses that can lead to a higher baseline ROS level, and they may respond more sensitively to nanoparticles treatment that affect their oxidative stress balance. On the other hand, HSF cells, which are non-cancerous, may have more stable oxidative homeostasis and may not exhibit a significant ROS change following treatment^20–22^. This discrepancy suggests that the treatment with CaTiO_3_NPs might specifically affect ROS generation in cancer cells due to their altered cellular environment or increased susceptibility to oxidative stress, whereas normal HSF cells may not be as responsive to the same treatment in terms of ROS production.

For a more in-depth look at CaTiO_3_NPs genotoxicity and its implications, the expression level of apoptotic (p53 and Bax) and anti-apoptotic Bcl2 genes have been measured using qRT-PCR. Apoptosis is a programmed cell death and plays a crucial role in maintaining the overall health and balance of cells. When cells are no longer functioning properly or are damaged beyond repair, apoptosis is triggered to eliminate them in a controlled and orderly manner^23^. Exposure to CaTiO_3_NPs caused significant increases in apoptotic cell death only in human lung cancer A-549 cells as manifested by the concurrent marked upregulation in the expression level of apoptotic p53 and Bax genes and a significant decrease in the anti-apoptotic Bcl2 expression level noticed after 48 h of lung cancer A-549 cells treatment with CaTiO_3_NPs. Consistency with our results CaTiO_3_NPs exhibited a potent selective genotoxicity and apoptotic death towards cancerous breast MCF-7 cells^8^.

Ongoing with the results of alkaline Comet assay the demonstrated apoptosis of treated lung cancer A-549 cells may result from CaTiO_3_NPs induced DNA breaks because DNA breakages are very dangerous stimulus cause disruption of the genomic integrity and trigger apoptotic cell death^24–26^. On the other hand the non- remarkable changes in the expression level of apoptotic and anti-apoptotic genes detected after treatment of normal HSF cells with CaTiO_3_NPs confirmed the non-genotoxicity of these nanoparticles towards normal HSF cells in consistence with the recent study of Mohamed et al.^9^, that demonstrated that CaTiO_3_NPs was non-genotoxic to normal HSF cells since non-significant changes were noticed in the genomic DNA integrity and expression level of apoptotic and anti-apoptotic genes’ expression after 72 h of normal HSF cells exposure to calcium titanate nanoparticles.

## Conclusion

In this study, exposure to CaTiO_3_NPs resulted in a low cytotoxic effect on both normal HSF and cancerous A-549 lung cells, with the IC50 values exceeding 1000 µg/ml for both cell types. Despite the relatively low cytotoxicity, CaTiO_3_NPs was non-genotoxic to normal HSF cells, whereas they selectively induced genotoxicity in cancerous A-549 lung cells. This selective genotoxicity was mediated by the excessive ROS generation, which led to genomic DNA damage and alterations in the expression of apoptotic and anti-apoptotic genes in lung cancer cells. These findings suggest that CaTiO_3_NPs have promising potential for targeted cancer therapy, specifically for lung cancer, where they can selectively induce oxidative stress and genotoxicity in cancer cells while sparing normal cells. However, further investigations re needed to better understand the molecular mechanisms underlying this selective toxicity, and to assess the therapeutic potential of CaTiO_3_NPs in human lung cancer cell lines and potentially other types of cancer.

## Data Availability

The datasets used and/or analyzed during the current study available from the corresponding author on reasonable request.
